# Effects of different fibrinogen concentrations on blood loss and coagulation parameters in a pig model of coagulopathy with blunt liver injury

**DOI:** 10.1186/cc8960

**Published:** 2010-04-14

**Authors:** Oliver Grottke, Till Braunschweig, Dietrich Henzler, Mark Coburn, Rene Tolba, Rolf Rossaint

**Affiliations:** 1 Department of Anaesthesiology, RWTH Aachen University Hospital Pauwelsstrasse 30, D-52074 Aachen, Germany; 2 Institute for Laboratory Animal Science, RWTH Aachen University Hospital, Pauwelsstrasse 30, D-52074 Aachen, Germany; 3 Department of Pathology, RWTH Aachen University Hospital, Pauwelsstrasse 30, D-52074 Aachen, Germany; 4 Department of Anaesthesia and Division of Critical Care, Dalhousie University Halifax, Queen Elisabeth II Health Sciences Center, 10 West Victoria, 1276 South Park St., Halifax, NS, B3H 2Y9, Canada

## Abstract

**Introduction:**

The early application of fibrinogen could potentially reverse haemodilution-induced coagulopathy, although the impact of varying concentrations of fibrinogen to reverse dilutional coagulopathy has not been studied *in vivo*. We postulated that fibrinogen concentration is correlated with blood loss in a pig model of coagulopathy with blunt liver injury.

**Methods:**

Coagulopathy was induced in 18 anaesthetized pigs (32 ± 1.6 kg body weight) by replacing 80% of blood volume with hydroxyethylstarch 130/0.4 and Ringer's lactated solution, and re-transfusion of erythrocytes. Animals were randomly assigned to receive either 70 mg kg^-1 ^(F-70) or 200 mg kg^-1 ^(F-200) fibrinogen or placebo before inducing blunt liver injury using a force of 225 ± 26 Newton. Haemodynamics, coagulation parameters and blood loss were monitored for 2 hours. After death, histological examination of internal organs was performed to assess the presence of emboli and the equality of liver injury.

**Results:**

Plasma dilution caused severe coagulopathy. Measured by thromboelastography fibrinogen restored coagulation dose-dependently. Total blood loss was significantly lower and survival better in both fibrinogen groups as compared to controls (*P *< 0.05). Between the F-70 (1317 ± 113 ml) and the F-200 group (1155 ± 232 ml) no significant difference in total blood loss could be observed, despite improved coagulation parameters in the F-200 group (*P *< 0.05). Microscopy revealed even injury pattern and no (micro) thrombi for either group.

**Conclusions:**

Restoring fibrinogen with 70 or 200 mg kg^-1 ^after severe dilutional coagulopathy safely improved coagulation and attenuated blood loss after experimental blunt liver trauma. The higher dosage of fibrinogen was not associated with a further reduction in blood loss.

## Introduction

Traumatised and surgical patients with massive haemorrhage are predisposed to develop coagulopathy, as a result of multiple mechanisms including acidosis, hypothermia, anaemia, hyperfibrinolysis and hypotension-induced inflammation, as well as consumption and dilution of coagulation factors [1]. Dilutional coagulopathy may occur after massive blood loss, as crystalloid and colloid solutions are infused for fluid resuscitation. The degree of coagulopathy depends on the type and volume of the fluids infused [2]. Resuscitation with colloid plasma expanders may lead to a functional fibrinogen deficiency by abnormal fibrin polymerisation, which can be reversed by exogenous fibrinogen [3]. Furthermore, fibrinogen concentrations of less than 100 mg dL^-1 ^may occur before other coagulation factors are diluted [4]. The early decrease of fibrinogen levels has led to the hypothesis that fibrinogen is a key factor for reversing haemodilution induced coagulopathy. Fresh frozen plasma (FFP), cryoprecipitate and fibrinogen may be substituted to restore low concentrations of fibrinogen. FFP contains all coagulation factors and is recommended according to international guidelines to be used either in massive bleeding or if a prolongation of prothrombin time (PT) activated thromboplastin time of more than 1.5 times is accompanied by signs of microvascular bleeding [5]. However, to restore coagulation with FFP and increase low levels of fibrinogen efficiently, large volumes of FFP are needed. Other drawbacks of FFP transfusion include immunological reactions such as transfusion-related lung injury, anaphylaxis and haemolysis in cases of ABO incompatibility [6]. Alternatively, cryoprecipitate might be used to raise critical levels of fibrinogen, containing factor VIII, fibrinogen, fibronectin, von Willebrand factor and factor XIII [7]. A dose of around 10 single bags of cryoprecipitate derived from units of whole blood typically raises the plasma fibrinogen level by up to 60 to 100 mg dL^-1^. However, due to the risk of blood-borne pathogen transmission, the use of cryoprecipitate for this indication is discussed critically. In respect to FFP and cryoprecipitate pasteurised fibrinogen is virus inactivated. In a pioneering study Fries and colleagues could demonstrate that the early application of 250 mg kg^-1 ^fibrinogen reversed haemodilution-induced coagulopathy and reduced blood loss in a porcine model of liver injury [8]. Subsequent studies confirmed these findings and also showed a dose-dependent effect of fibrinogen substitution on thromboelastometry variables *in vitro *[9-11]. However, as *in vitro *studies are performed under low shear conditions, the effects of the interaction with vascular endothelium were not investigated and relevant clinical outcomes in relation to increasing doses of fibrinogen, such as blood loss and survival rate, remained unknown [12,13]. Therefore this study investigated a possible dose-dependent effect of fibrinogen to reverse haemodilution *in vivo *in a model of blunt liver injury. Primary endpoints of this study included blood loss and survival time, secondary endpoints were improvement in coagulation tests including thromboelastometry and the evaluation of adverse events.

## Materials and methods

### Ethics and anaesthesia

All experiments were performed in accordance with the German legislation governing animal studies following The *Principles of Laboratory Animal Care *[14]. Official permission for this study was granted from the governmental animal care and use office (Landesamt für Natur, Umwelt und Verbraucherschutz Nordrhein-Westfalen, Recklinghausen, Germany).

Before surgery, pigs were housed in ventilated rooms and allowed to acclimatise to their surroundings for a minimum of five days. Animals were fasted overnight before surgical procedure, with water allowed *ad libitum*.

Eighteen German male land-race pigs, weighing (mean ± standard deviation (SD)) 32 ± 1.6 kg received an intramuscular injection of 4 mg kg^-1 ^azaperone (Stresnil™, Janssen, Neuss, Germany) as pre-medication. Anaesthesia was induced by an intravenous injection of 3 mg kg^-1 ^propofol (Disoprivan^®^, Astra Zeneca, Wedel, Germany) followed by orotracheal intubation. The animals were ventilated with 20 to 26 breaths min^-1 ^and a tidal volume of 10 mL kg^-1 ^to keep the end-tidal partial pressure of carbon dioxide (pCO_2_) between 36 and 42 mmHg. The inspiratory oxygen fraction was 1.0 during haemodilution and reduced to 0.4 afterwards. Anaesthesia was maintained with isoflurane at end-tidal concentrations of 1% to 1.2% and continuous infusion of fentanyl at 3 μg kg^-1 ^h^-1^. Ringer's lactated solution (RL) was infused at 4 mL kg^-1^h^-1 ^at first and increased to 8 mL kg^-1^h^-1^after laparotomy until infliction of trauma. Body temperature was maintained over the entire experiment (36.5 to 37.0°C) with a warming blanket.

Monitoring included electrocardiography, tail pulse oximetry, temperature and arterial and central venous pressure by femorally introduced catheters connected to a standard anaesthesia monitor (AS/3, Datex Ohmeda, Helsinki, Finland).

### Surgical preparation and haemodilution

Two 8.5 Fr catheters were surgically implanted in the right and left jugular veins for volume substitution and insertion of a pulmonary artery catheter. A splenectomy was performed under neuromuscular blockage with pancuronium (0.2 mg kg^-1 ^intravenous). To compensate for blood loss associated with the splenectomy, a bolus of warmed RL three times the weight of the spleen was administered. To achieve comparable low concentrations of fibrinogen, intravascular volume was diluted by replacing approximately 80% of the estimated blood volume [15] by hydroxyethylstarch 130/0.4 (Voluven^®^, Fresenius, Bad Homburg, Germany) with a maximum dose of 50 ml kg^-1 ^and RL in a ratio of 1:1.2 to 1.5. The collected blood was processed (Cell Saver 5^®^, Haemonetics, Munich, Germany) and the red cells were re-transfused before trauma infliction to avoid early death from severe anaemia.

### Fibrinogen substitution and liver injury

Six animals each were randomised to receive normal saline solution (controls), 70 mg kg^-1 ^fibrinogen (group F-70; fibrinogen: Haemocompletan^®^, CSL Behring, Marburg, Germany), or 200 mg kg^-1 ^(group F-200) fibrinogen. Subsequently, a grade III blunt liver injury [16] was inflicted as described before using a custom-made instrument [17]. Briefly, the liver was gently retracted to allow adequate exposure. The base of the plate was positioned beneath the right middle lobe. The injury was induced by one-time clamping of the instrument through the parenchyma with a force of 225 ± 26 Newton (N). The force of injury was analysed in real-time and the signal was displayed in a visual programming environment (LabView 8.8, National Instruments, Austin, TX, USA) after amplifying (VG140, ATR Industrie-Elektronik, Krefeld, Germany) and digitizing the force signal (NI USB-6009, National Instruments, Austin, TX, USA). The time of registration was set to 500 msec. In all cases the injury was inflicted by the same investigator (OG), being also blinded to the experimental group.

After liver injury, the abdomen was closed with staples and further manipulations were avoided. Five minutes after injury all animals received 4 mL kg ^-1 ^min^-1 ^of RL given over eight minute. Afterwards, the rate was set to 25 mL kg^-1 ^h^-1 ^until the end of the experiment. The observation period ended at 120 minutes after injury. Pulseless electrical activity, a mean arterial pressure of less than 10 mmHg and an end-tidal PCO_2 _of less than 10 mmHg were defined as death. Animals surviving for more than two hours were killed with fentanyl, propofol and potassium chloride. Immediately after death, the abdomen was reopened, the vena cava was clamped cranial to the liver and the intraperitoneal blood was collected to determine the total blood loss post-injury. After that, internal organs (lungs, heart, liver and kidneys) were removed and prepared for histological examination.

### Blood sampling and analytical methods

Blood was collected and arterial blood gas analysis were performed 10 minutes after splenectomy ('baseline'), at the end of haemodilution ('haemodilution'), after fibrinogen substitution ('fibrinogen') and 120 minutes after liver injury ('trauma') or immediately following death, whichever occurred first. Haemoglobin concentration, pH value, partial pressure of oxygen (pO_2_) and carbon dioxide (pCO_2_) were measured with a blood gas analyser (ABL500, Radiometer, Copenhagen, Denmark). Prothrombin time (PT), activated partial thromboplastin time (aPTT) and fibrinogen concentrations were determined by standard laboratory methods using the appropriate tests from Dade Behring (Marburg, Germany) on a coagulometer (KC4, Baxter, Newbury, UK). Thrombin-antithrombin (TAT) complexes were quantified by ELISA (TAT, Dade Behring, Germany). A coagulation analyser (ROTEM^®^, Pentapharm, Munich, Germany) was used for thrombelastometry with the EXTEM^® ^assay according to the manufacturer's instructions. The following parameters were obtained: clot formation time (CFT in seconds: reflects the coagulation time until 20 mm of amplitude are reached), maximum clot firmness (MCF in mm: reflects the strength of a resulting clot) and the α-angle (in degree: shows the rate of fibrin polymerisation).

### Pathological examination

All investigated internal organs, such as the lungs, the heart, liver and the kidneys, were removed after death and directly fixed in 10% buffered formalin. Injured parts of the liver were cut into 3 mm thick slices. Only areas of maximum depth of injury and most severe vessel rupture were chosen for further histological examination. In addition representative tissue sections of all four organs were processed to explore for thrombotic events. All samples were embedded in paraffin and stained by H&E and a standard Elastica-van Gieson protocol for histological examination under light microscopy (Eclipse 50i, Nikon, Duesseldorf, Germany). Secondary, suspect sections of lung and liver tissues were immunostained for fibrinogen and von Willebrand factor. A polyclonal rabbit antihuman fibrinogen antibody (DAKO A0080, polyclonal rabbit, DAKO, Glostrup, Denmark) and polyclonal rabbit von Willebrand factor VIII antibody (DAKO, A0082, polyclonal rabbit) were used at a concentration of 1:100. For staining, the ABC Vectastain universal kit (Vector Laboratories, Burlingame, CA, USA) and haematoxylin as counterstain was used. A blinded pathologist subsequently assessed the degree of injury in the liver and of fibrin deposition in vessels and microthrombi formation in all organs.

### Statistical analysis

Data are presented as mean ± SD (SPSS V16, Chicago, IL, USA). Normal distribution of parameters was shown on the interpretation of Q-Q plots and histograms. Differences between groups were analysed with a one-way analysis of variance (ANOVA) with Scheffe's *post hoc *test and Games Howell for multiple comparisons, respectively. A repeated measures ANOVA was applied to analyse the influence of dilution and treatment substitution over time using Scheffe's *post hoc *and Games Howell tests.

Non-parametric distributed parameters of thromboelastometry were analysed using Kruskal-Wallis H-test and Bonferroni-Dunn tests for multiple comparisons. Data are presented in box plots. Data on survival were analysed by the log-rank test. Statistical tests were performed two-tailed and the level of significance was defined as *P *< 0.05.

## Results

### Baseline measurements and coagulation parameters after haemodilution

**Figure 1:**
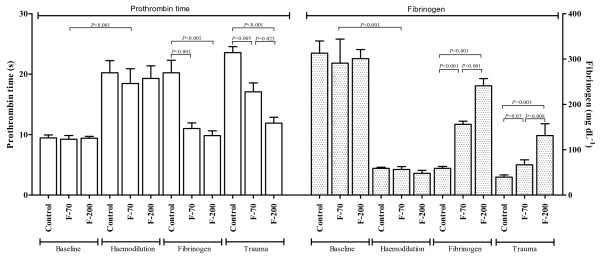
**Prothrombin time and fibrinogen concentrations at baseline, after haemodilution, fibrinogen substitution (fibrinogen) and trauma**. Data presented as mean ± standard deviation.

**Figure 2:**
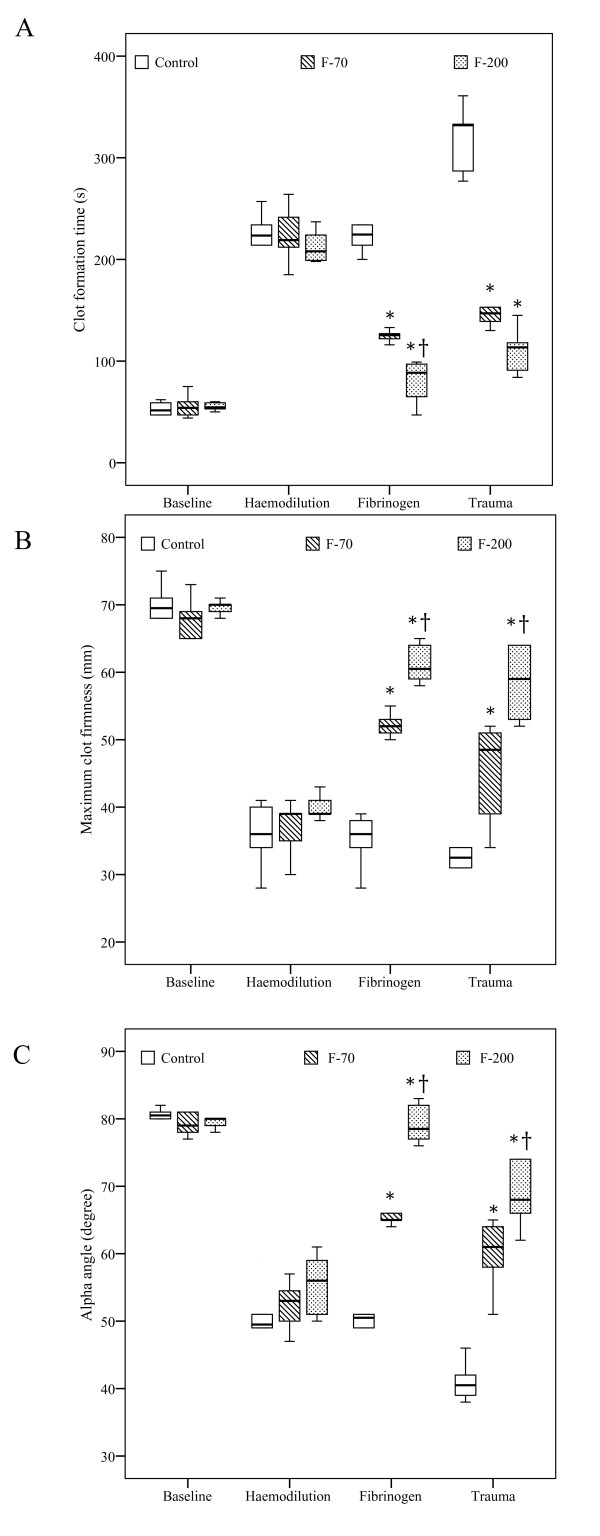
**Thromboelastometry parameters**. **(a)** Clot formation time, **(b)** Maximum clot firmness and **(c)** α-angle at various time points including baseline, after haemodilution, fibrinogen substitution (fibrinogen) and trauma. Results are shown as box plots (minimum, first quartile, median, third quartile, maximum). *P < 0.05 vs. control; †*P *< 0.05 vs. F-70.

Baseline parameters were comparable between groups (Tables 1 and 2). The dilution caused a significant coagulopathy and a drop in platelets. PT increased from 9.3 ± 0.7 seconds to 19 ± 2 seconds (pooled data) whereas fibrinogen concentrations decreased from 301 ± 36 mg dL^-1 ^to 54 ± 7 mg dL^-1 ^(*P *< 0.001; Figure 1). Coagulopathy was adequately detected by significant findings in thromboelastometry (*P *< 0.001; Figure 2). No clinical signs of coagulopathy, such as oozing from insertion sites or mucosal bleeding, were observed in any group.

**Table 1:** Laboratory parameters (mean ± standard deviation). Parameters included in the table are haemoglobin, platelet count (PLT), prothrombin time (PT), activated partial thromboplastin time (aPTT) and thrombin-AT complex (TAT) at baseline, after haemodilution, after fibrinogen substitution (fibrinogen) at the end of the observation period (trauma)

	Baseline	Haemodilution	Fibrinogen	Trauma
Haemoglobin (g L^-1^)				
Control	8.2 ± 0.5	7.5 ± 0.6	7.8 ± 0.4	3.7 ± 0.4
F-70	8.1 ± 0.4	7.7 ± 0.4	7.9 ± 0.6	4.1 ± 0.3
F-200	8.0 ± 0.2	8.0 ± 0.6	8.0 ± 0.5	4.5 ± 1.7
PLT (10^3 ^μL^-1^)				
Control	268 ± 35	85 ± 8	84 ± 11	46 ± 4
F-70	263 ± 44	87 ± 10	87 ± 13	54 ± 14
F-200	294 ± 41	94 ± 11	95 ± 12	71 ± 10*
aPTT (s)				
Control	12 ± 1	22 ± 5	22 ± 5	27 ± 6
F-70	11 ± 2	24 ± 6	22 ± 5	28 ± 3
F-200	11 ± 1	23 ± 2	20 ± 2	21 ± 5
TAT (μg L^-1^)				
Control	10.4 ± 3.2	11.3 ± 3.5	10.4 ± 3.0	17.4 ± 6.7
F-70	8.0 ± 3.0	8.1 ± 1.6	9.1 ± 1.5	12.9 ± 5.5
F-200	10.2 ± 2.8	8.5 ± 5.7	8.4 ± 2.9	12.3 ± 2.0

*P < 0.005 F-200 vs. control.

**Table 2:** Haemodynamic parameters (mean ± standard deviation). Parameters included in the table are heart rate (HR), mean arterial pressure (MAP), central venous pressure (CVP), mean pulmonary pressure (MPAP) and cardiac output (CO) at baseline (after splenectomy), after fibrinogen substitution (fibrinogen), haemodilution, five minutes after trauma and at the end of the observation period (trauma)

	Baseline	Haemodilution	Fibrinogen	5 min after Trauma	Trauma
HR (beats min^-1^)					
Control	83 ± 15	99 ± 11	99 ± 10	115 ± 12	155 ± 22
F-70	86 ± 12	92 ± 13	84 ± 11	110 ± 10	146 ± 15
F-200	87 ± 13	81 ± 18	82 ± 12	127 ± 14	175 ± 13
MAP (mmHg)					
Control	79 ± 10	78 ± 6	75 ± 5	41 ± 4	13 ± 1
F-70	75 ± 9	75 ± 8	76 ± 6	39 ± 3	29 ± 7*
F-200	83 ± 11	77 ± 5	81 ± 5	45 ± 5	36 ± 4*
CVP (mmHg)					
Control	8 ± 2	7 ± 2	7 ± 2	4 ± 1	1 ± 1
F-70	8 ± 1	8 ± 2	8 ± 1	5 ± 1	4 ± 0.5*
F-200	9 ± 2	8 ± 3	8 ± 1	5 ± 2	6 ± 1*
MPAP (mmHg)					
Control	20 ± 1	20 ± 2	19 ± 3	14 ± 1	7 ± 2
F-70	18 ± 2	20 ± 3	20 ± 3	13 ± 2	9 ± 1
F-200	17 ± 2	18 ± 2	21 ± 3	12 ± 1	12 ± 2*
CO (L min^-1^)					
Control	4.4 ± 0.5	4.0 ± 0.7	3.7 ± 0.7	2.5 ± 0.2	1.2 ± 0.5
F-70	3.7 ± 0.7	3.4 ± 0.4	3.4 ± 0.5	2.6 ± 0.3	2.3 ± 0.4*
F-200	4.5 ± 0.8	3.6 ± 0.9	3.6 ± 0.6	2.2 ± 0.2	2.5 ± 0.5*

*P < 0.005 vs. control.

### Coagulation parameters after fibrinogen substitution and after injury

Fibrinogen substitution significantly increased the concentrations of fibrinogen in the intervention groups (F-70: 148 ± 7 mg dL^-1^; F-200: 237 ± 17 mg dL^-1^). Although PT decreased equally in both intervention groups (Figure 1), the decrease in CFT and the increases in MCF and α-angle were dose dependent (Figure 2).

After haemodilution and liver injury fibrinogen concentrations decreased in all groups over time. However, in the F-200 group, fibrinogen concentration was significantly higher (131 ± 26 mg dL^-1^) and PT lower (11 ± 1 seconds) than in F-70 group (fibrinogen: 67 ± 11 mg dL^-1^, PT 17 ± 2 seconds). Corresponding results were obtained by thromboelastometry (Figure 2), with controls showing consistently lowest (MCF, α-angle), respectively highest values (CFT).

The aPTT was significantly prolonged after dilution, without significant differences between groups. Similarly, TAT complexes increased in all groups without significant differences between groups (Table 1). Concentrations of D-Dimer were below 500 μg l^-1 ^at all times (data not shown).

### Haemodynamics and blood loss

No differences in haemodynamics were observed between groups until liver injury (Table 2). Following liver injury all animals developed haemorrhagic shock. Blood loss after liver injury was highest in the control group (1803 ± 248 ml; *P *< 0.05), followed by the F-70 (1317 ± 113 ml) and F-200 (1155 ± 232 ml) groups. The difference in blood loss between the intervention groups was not significant (*P *= 0.205). Mean arterial pressure and cardiac output were significantly lower in controls than in the intervention groups.

**Figure 3:**
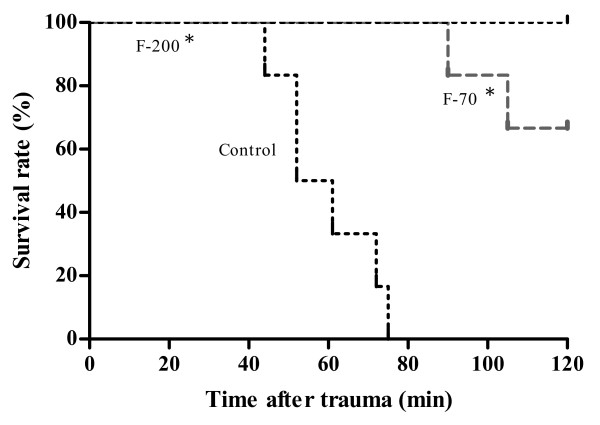
**Data of survival are presented as a Kaplan-Meier curve**. *P < 0.05 vs. control.

All animals in the control group died before the end of the observation period, with a survival time of 59 ± 12 minutes (Figure 3). All animals in the F-200 group survived, whereas two out of six animals (33%) of the F-70 group died before the end of the observation time (*P *= 0.138).

### Histopathological analysis

Macroscopical and histological evaluation by immunostaining with von Willebrand factor of injured liver sections revealed an equal tissue damage as well as comparable laceration of venous vessels of a maximum of 3 to 4 mm. No evidence for thrombus formation or microthrombi was found in the H&E and fibrinogen stain of kidney, heart or lung tissues.

## Discussion

In this *in vivo *study we could demonstrate a dose-dependent effect of fibrinogen to reverse haemodilution-induced coagulopathy. Increasing concentrations of fibrinogen resulted in a further improvement of clot formation and clot firmness. Blood loss after liver injury was significantly lower in the fibrinogen groups as compared with controls, but there was no difference between substituting 70 or 200 mg kg^-1 ^fibrinogen in regards to blood loss or survival.

After haemodilution, coagulation was severely impaired as shown by prolonged PT, clot formation and an overall reduction in clot firmness. To meet a plasma fibrinogen concentration below the threshold of international recommendations (> 80 to 100 mg dL^-1^) [5], the degree of haemodilution was set to achieve a fibrinogen concentration of approximately 50 mg dL^-1^. Although it is well known that colloids may interfere with concentrations of fibrinogen determined by the Clauss method [18], the prolongation of clot formation and decreased clot strength confirmed the haemodilution induced coagulopathy in our study.

In concordance with several *in vitro *studies we could show that increasing concentrations of fibrinogen dose dependently improved clot formation (lower CFT and higher α-angle) and clot strength (increase of MCF) using the EXTEM^® ^assay [9-11]. Although the FIBTEM^® ^assay specifically attributes the impact of fibrinogen/fibrin on clot strength by inhibiting platelets through cytochalasin-D [19], the FIBTEM^® ^assay cannot be reliable used with porcine blood [20]. The observed improvement on clot strength after fibrinogen substitution is most likely explained by its binding to GIIb/IIIa receptors, as the platelet count did not significantly vary after haemodilution and fibrinogen substitution. The abundant number of approximately 40,000 to 50,000 GIIb/IIIa receptors per activated platelet allows binding of large amounts of fibrinogen [21]. This theory is supported by a possible compensating role of fibrinogen with the presence of thrombocytopenia [22,23]. However, even at very high doses of exogenous fibrinogen, clot strength did not reach baseline values, probably because of reduced levels of other coagulation factors such as FXIII [24-26]. In contrast, prolonged PTs were reversed after fibrinogen provision, but this effect was not dose dependent. As the PT only reflects 5% of the whole coagulation process the substitution of 70 mg kg^-1 ^of fibrinogen already normalised PT down to almost baseline values despite altered thromboelastometry variables [27]. Our observation confirms that the sensitivity of PT is insufficient to guide haemostatic therapy in haemodilution-induced coagulopathy [28]. A recommendation by the Society of Thoracic Surgeons therefore suggests guiding haemostatic therapy by point-of-care testing rather than by plasma-based coagulation assays [29].

Following trauma, haemodilution and shock decreased both the clot formation and clot strength in all animals. This effect is due to the loss, consumption and dilution of coagulation factors. However, at the end of the observation period clot strength and fibrinogen concentration were still higher in the F-200 group as compared with the F-70 group, but not associated with a further reduction in blood loss. Thus the endogenous potential of procoagulant activators was sufficient to activate fibrinogen and to terminate bleeding. The comparable blood loss between the F-70 and F-200 group and the similar decrease of the mean concentration of fibrinogen indicates that the limiting factor determining the time to haemostasis was rather restricted by the concentration of fibrinogen than its activation. Although thrombin generation and clot formation have been shown to be decreased at a plasma dilution of more than 40% [30], the residual thrombin is usually sufficient to cleave fibrinogen. However, trauma-induced blood loss and haemodilution further reduced the rate of thrombin generation that is crucial to achieve sufficient haemostasis. Due to the short half-life time of FXa and thrombin [31] clinical situations with dilutional coagulopathy and active bleeding might also require the additional substitution of procoagulant factors [32].

Although some recent studies indicate a potential protective effect of higher levels of fibrinogen to reduce blood loss [33-36], fibrinogen is rarely used as monotherapy but as an adjunct in clinical situations with life-threatening bleeding. Thus the substitution of fibrinogen may be a reasonable approach to reduce the use of allogenic blood products, but the efficacy of fibrinogen substitution may also be enhanced by the concomitant application of other coagulation factors.

There is some concern that substitution of fibrinogen may enhance the risk for thromboembolic events. This risk may be aggravated by the concomitant application of other haemostatics, such as antifibrinolytics. Although some studies indicate an association between chronic elevation of fibrinogen and an increased risk for cardiovascular events [37], a systematic review about the safety of fibrinogen substitution in a situation of deficit showed a low thrombogeneity [38]. Our results are consistent with these studies, as we could not detect (micro) vascular thrombosis or hypercoagulability after fibrinogen substitution.

Some limitations do apply. Despite the application of up to 200 mg kg^-1 ^fibrinogen, thromboelastometry variables were not restored to baseline values. The provision of other coagulation factors, such as FXIII might have shown different results. The experimental setup required inducing the haemodilution and fibrinogen application before the infliction of trauma. It does not exactly mirror a clinical situation, where coagulopathy occurs after trauma. Although we mimicked a blunt liver injury, we allowed free bleeding after the injury, which is somewhat similar to a clinical situation with penetrating trauma. Further, the observation time of this study was limited to only two hours, which prevented the study of further treatment effects or possible physiological compensation mechanisms. However, all of the control animals had died within this observation period, which demonstrates a clear treatment effect. In addition, the induction of injury was performed in anaesthetised healthy pigs. Thus, the physiological response to such things as pain and inflammation may have additional effects on haemostasis, which are not reflected in our model.

Finally, there is a great debate about the ideal resuscitation fluid, which has been recently addressed in a Cochrane systematic review [39]. It would be way beyond the scope of this study to discuss the implications of the fluids needed to maintain haemodynamic stability in our pig model of severe dilutional coagulopathy. Both crystalloid and colloid solutions have manifold influence on the coagulation system, inflammatory responses and organ function. Our model of a combination of hydroxyethylstarch and crystalloid represents the current concept at our institution, and probably of many other centres, for resuscitation of haemorrhagic shock, until the optimum resuscitation strategy has been identified.

## Conclusions

In summary, dilutional coagulopathy could be reversed by the early administration of exogenous fibrinogen in the absence of severe anaemia. Higher doses of fibrinogen correlated with improved parameters of thromboelastometry and may be a reasonable approach to reduce the use of FFP, platelet concentrate and red blood cells as these allogenic blood products are associated with various adverse outcomes. However, the results of our study also show that substituting fibrinogen concentration to values of more than 150 mg dL^-1 ^had no additional effect on clinical relevant endpoints in this specific animal model, if no other coagulation factors or thrombocytes were transfused. Thus, future clinical studies should address the question of optimum level of fibrinogen in combination with the replacement of other clotting factors, timing of fibrinogen substitution and patient selection.

## Key messages

• We could demonstrate that restoring fibrinogen with 70 or 200 mg kg^-1 ^after severe dilutional coagulopathy dose dependently improved coagulation parameters as shown by thromboelastometry variables.

• Although blood loss after liver injury was significantly lower in the fibrinogen groups as compared with controls, there was no difference between substituting 70 or 200 mg kg^-1 ^fibrinogen in regard to blood loss or survival. Therefore substituting fibrinogen concentration to values above 150 mg dL^-1 ^had no additional effect on clinical relevant endpoints in this specific animal model.

• As no (micro) vascular thrombosis or hypercoagulability was observed, the early application of fibrinogen might be a safe approach to restore critical concentrations of fibrinogen and may reduce the need for the transfusion of allogenic blood products.

## Abbreviations

ANOVA: analysis of variance; aPTT: activated partial thromboplastin time; CFT: clot formation time; ELISA: enzyme-linked immunosorbent assay; FFP: fresh frozen plasma; H&E: haematoxylin and eosin; MCF: maximum clot firmness; pCO_2_: partial pressure of carbon dioxide; pO_2_: partial pressure of oxygen; PT: prothrombin time; RL: Ringer's lactated solution; SD: standard deviation; TAT: thrombin-antithrombin.

## Competing interests

RR has received honoraria for lectures and consultancy from CSL Behring, Germany. Fibrinogen (Haemocompletan^®^) was provided by CSL Behring, Marburg, Germany for the current study, but there was not financial support. The authors declare that they have no other competing interests.

## Authors' contributions

OG conceived and conducted the experimental laboratory work, performed the statistical analysis and drafted the manuscript. RR participated in the study design and coordination and helped to draft the manuscript. TB, MC, and RT helped to perform the study and draft the manuscript. DH draft the manuscript. All authors read and approved the final manuscript.

## References

[B1] Hess JR Brohi K Dutton RP Hauser CJ Holcomb JB Kluger Y Mackway-Jones K Parr MJ Rizoli SB Yukioka T Hoyt DB Bouillon B The coagulopathy of trauma: a review of mechanisms J Trauma 2008 65 748 754 10.1097/TA.0b013e3181877a9c 18849786

[B2] Mittermayr M Streif W Haas T Fries D Velik-Salchner C Klingler A Innerhofer P Hemostatic changes after crystalloid or colloid fluid administration during major orthopedic surgery: the role of fibrinogen administration Anesth Analg 2007 105 905 917 10.1213/01.ane.0000280481.18570.27 17898365

[B3] Fenger-Eriksen C Anker-Møller E Heslop J Ingerslev J Sørensen B Thrombelastographic whole blood clot formation after ex vivo addition of plasma substitutes: improvements of the induced coagulopathy with fibrinogen concentrate Br J Anaesth 2005 94 324 329 10.1093/bja/aei052 15608046

[B4] Hiippala ST Myllylä GJ Vahtera EM Hemostatic factors and replacement of major blood loss with plasma-poor red cell concentrates Anesth Analg 1995 81 360 365 10.1097/00000539-199508000-00026 7542432

[B5] Spahn DR Cerny V Coats TJ Duranteau J Fernández-Mondéjar E Gordini G Stahel PF Hunt BJ Komadina R Neugebauer E Ozier Y Riddez L Schultz A Vincent JL Rossaint R Task Force for Advanced Bleeding Care in Trauma Management of bleeding following major trauma: a European guideline Crit Care 2007 11 R17 10.1186/cc5686 17298665 PMC2151863

[B6] O'Shaughnessy DF Atterbury C Bolton Maggs P Murphy M Thomas D Yates S Williamson LM British Committee for Standards in Haematology, Blood Transfusion Task Force Guidelines for the use of fresh-frozen plasma, cryoprecipitate and cryosupernatant Br J Haematol 2004 126 11 28 10.1111/j.1365-2141.2004.04972.x 15198728

[B7] Callum JL Karkouti K Lin Y Cryoprecipitate: the current state of knowledge Transfus Med Rev 2009 23 177 188 10.1016/j.tmrv.2009.03.001 19539873

[B8] Fries D Krismer A Klingler A Streif W Klima G Wenzel V Haas T Innerhofer P Effect of fibrinogen on reversal of dilutional coagulopathy: a porcine model Br J Anaesth 2005 95 172 177 10.1093/bja/aei160 15923269

[B9] Fries D Innerhofer P Reif C Streif W Klingler A Schobersberger W Velik-Salchner C Friesenecker B The effect of fibrinogen substitution on reversal of dilutional coagulopathy: an in vitro model Anesth Analg 2006 102 347 351 10.1213/01.ane.0000194359.06286.d4 16428520

[B10] Haas T Fries D Velik-Salchner C Reif C Klingler A Innerhofer P The in vitro effects of fibrinogen concentrate, factor XIII and fresh frozen plasma on impaired clot formation after 60% dilution Anesth Analg 2008 106 1360 1365 10.1213/01.ane.0b013e3181684339 18420845

[B11] Bolliger D Szlam F Molinaro RJ Rahe-Meyer N Levy JH Tanaka KA Finding the optimal concentration range for fibrinogen replacement after severe haemodilution: an in vitro model Br J Anaesth 2009 102 793 799 10.1093/bja/aep098 19420005

[B12] Ruggeri ZM The role of von Willebrand factor and fibrinogen in the initiation of platelet adhesion to thrombogenic surfaces Thromb Haemost 1995 74 460 463 8578507

[B13] Ruggeri ZM Mechanisms initiating platelet thrombus formation Thromb Haemost 1997 78 611 616 9198225

[B14] Guide for the care and use of laboratory animals Institute of Laboratory Animal Resources, Commission on Life Sciences, National Research Council 1996 7 Washington DC: National Academy Press

[B15] Bush JA Jesen WN Cartwright GE Wintrobe MM Blood volume studies in normal and anemic swine Am J Physiol 1955 181 9 14 14376559 10.1152/ajplegacy.1955.181.1.9

[B16] Moore EE Cogbill TH Jurkovich GJ Shackford SR Malangoni MA Champion HR Organ injury scaling: spleen and liver (1994 revision) J Trauma 1995 38 323 324 10.1097/00005373-199503000-00001 7897707

[B17] Grottke O Braunschweig T Philippen B Gatzweiler KH Gronloh N Staat M Rossaint R Tolba R A new model for blunt liver injuries in the swine Eur Surg Res 2010 44 65 73 10.1159/000265053 19996600

[B18] Hiippala ST Dextran and hydroxyethyl starch interfere with fibrinogen assays Blood Coagul Fibrinolysis 1995 6 743 746 10.1097/00001721-199512000-00008 8825225

[B19] Lang T Toller W Gütl M Mahla E Metzler H Rehak P März W Halwachs-Baumann G Different effects of abciximab and cytochalasin D on clot strength in thrombelastography J Thromb Haemost 2004 2 147 153 10.1111/j.1538-7836.2004.00555.x 14717978

[B20] Velik-Salchner C Schnürer C Fries D Müssigang PR Moser PL Streif W Kolbitsch C Lorenz IH Normal values for thrombelastography (ROTEM) and selected coagulation parameters in porcine blood Thromb Res 2006 117 597 602 10.1016/j.thromres.2005.05.015 15985284

[B21] Wagner CL Mascelli MA Neblock DS Weisman HF Coller BS Jordan RE Analysis of GPIIb/IIIa receptor number by quantification of 7E3 binding to human platelets Blood 1996 88 907 914 8704248

[B22] Velik-Salchner C Haas T Innerhofer P Streif W Nussbaumer W Klingler A Klima G Martinowitz U Fries D The effect of fibrinogen concentrate on thrombocytopenia J Thromb Haemost 2007 5 1019 1025 10.1111/j.1538-7836.2007.02481.x 17461931

[B23] Lang T Johanning K Metzler H Piepenbrock S Solomon C Rahe-Meyer N Tanaka KA The effects of fibrinogen levels on thromboelastometric variables in the presence of thrombocytopenia Anesth Analg 2009 108 751 758 10.1213/ane.0b013e3181966675 19224779

[B24] Nielsen VG Gurley WQ Jr Burch TM The impact of factor XIII on coagulation kinetics and clot strength determined by thrombelastography Anesth Analg 2004 99 120 123 10.1213/01.ANE.0000123012.24871.62 15281516

[B25] Schroeder V Chatterjee T Kohler HP Influence of blood coagulation factor XIII and FXIII Val34Leu on plasma clot formation measured by thrombelastography Thromb Res 2001 104 467 474 10.1016/S0049-3848(01)00395-4 11755957

[B26] Nielsen VG Cohen BM Cohen E Effects of coagulation factor deficiency on plasma coagulation kinetics determined via thrombelastography: critical roles of fibrinogen and factors II, VII, X and XII Acta Anaesthesiol Scand 2005 49 222 231 10.1111/j.1399-6576.2005.00602.x 15715625

[B27] Negrier C Dargaud Y Bordet JC Basic aspects of bypassing agents Haemophilia 2006 12 48 52 10.1111/j.1365-2516.2006.01366.x 17123394

[B28] Kheirabadi BS Crissey JM Deguzman R Holcomb JB In vivo bleeding time and in vitro thrombelastography measurements are better indicators of dilutional hypothermic coagulopathy than prothrombin time J Trauma 2007 62 1352 1359 10.1097/TA.0b013e318047b805 17563647

[B29] Society of Thoracic Surgeons Blood Conservation Guideline Task Force Ferraris VA Ferraris SP Saha SP Hessel EA Haan CK Royston BD Bridges CR Higgins RS Despotis G Brown JR Society of Cardiovascular Anesthesiologists Special Task Force on Blood Transfusion Spiess BD Shore-Lesserson L Stafford-Smith M Mazer CD Bennett-Guerrero E Hill SE Body S Perioperative blood transfusion and blood conservation in cardiac surgery: the Society of Thoracic Surgeons and The Society of Cardiovascular Anesthesiologists clinical practice guideline Ann Thorac Surg 2007 83 S27 S86 10.1016/j.athoracsur.2007.02.099 17462454

[B30] Schols SE Feijge MA Lancé MD Hamulyák K ten Cate H Heemskerk JW van Pampus EC Effects of plasma dilution on tissue-factor-induced thrombin generation and thromboelastography: partly compensating role of platelets Transfusion 2008 48 2384 2394 10.1111/j.1537-2995.2008.01872.x 18673348

[B31] Jesty J Beltrami E Positive feedbacks of coagulation: their role in threshold regulation Arterioscler Thromb Vasc Biol 2005 25 2463 2469 10.1161/01.ATV.0000187463.91403.b2 16179597

[B32] Schols SE Meijden PE van der van Oerle R Curvers J Heemskerk JW van Pampus EC Increased thrombin generation and fibrinogen level after therapeutic plasma transfusion: relation to bleeding Thromb Haemost 2008 99 64 70 18217136 10.1160/TH07-07-0438

[B33] Charbit B Mandelbrot L Samain E Baron G Haddaoui B Keita H Sibony O Mahieu-Caputo D Hurtaud-Roux MF Huisse MG Denninger MH de Prost D PPH Study Group The decrease of fibrinogen is an early predictor of the severity of postpartum hemorrhage J Thromb Haemost 2007 5 266 273 10.1111/j.1538-7836.2007.02297.x 17087729

[B34] Karlsson M Ternström L Hyllner M Baghaei F Flinck A Skrtic S Jeppsson A Prophylactic fibrinogen infusion reduces bleeding after coronary artery bypass surgery. A prospective randomised pilot study Thromb Haemost 2009 102 137 144 19572078 10.1160/TH08-09-0587

[B35] Rahe-Meyer N Pichlmaier M Haverich A Solomon C Winterhalter M Piepenbrock S Tanaka KA Bleeding management with fibrinogen concentrate targeting a high-normal plasma fibrinogen level: a pilot study Br J Anaesth 2009 102 785 792 10.1093/bja/aep089 19411671 PMC2683341

[B36] Fenger-Eriksen C Jensen TM Kristensen BS Jensen KM Tønnesen E Ingerslev J Sørensen B Fibrinogen substitution improves whole blood clot firmness after dilution with hydroxyethyl starch in bleeding patients undergoing radical cystectomy: a randomized, placebo-controlled clinical trial J Thromb Haemost 2009 7 795 802 10.1111/j.1538-7836.2009.03331.x 19320829

[B37] Tatli E Ozcelik F Aktoz M Plasma fibrinogen level may predict critical coronary artery stenosis in young adults with myocardial infarction Cardiol J 2009 16 317 320 19653173

[B38] Dickneite G Pragst I Joch C Bergman GE Animal model and clinical evidence indicating low thrombogenic potential of fibrinogen concentrate (Haemocomplettan P) Blood Coagul Fibrinolysis 2009 in press 19710609 10.1097/MBC.0b013e32832da1c5

[B39] Dart AB Mutter TC Ruth CA Taback SP Hydroxyethyl starch (HES) versus other fluid therapies: effects on kidney function Cochrane Database Syst Rev 2010 CCD007594 20091640 10.1002/14651858.CD007594.pub2

